# Improved Fracture Toughness and Conversion Degree of Resin-Based Dental Composites after Modification with Liquid Rubber

**DOI:** 10.3390/ma13122704

**Published:** 2020-06-14

**Authors:** Krzysztof Pałka, Joanna Kleczewska, Emil Sasimowski, Anna Belcarz, Agata Przekora

**Affiliations:** 1 Faculty of Mechanical Engineering, Lublin University of Technology, Nadbystrzycka 36, 20-618 Lublin, Poland; e.sasimowski@pollub.pl; 2 Arkona Laboratorium Farmakologii Stomatologicznej, Nasutów 99C, 21-025 Niemce, Poland; Joanna@arkonadent.com; 3 Department of Biochemistry and Biotechnology, Medical University of Lublin, Chodźki 1, 20-093 Lublin, Poland; anna.belcarz@umlub.pl

**Keywords:** rubber toughening, dental material, fracture toughness, elasticity, degree of conversion, viscosity, cytotoxicity

## Abstract

There are many methods widely applied in the engineering of biomaterials to improve the mechanical properties of the dental composites. The aim of this study was to assess the effect of modification of dental composites with liquid rubber on their mechanical properties, degree of conversion, viscosity, and cytotoxicity. Both flow and packable composite consisted of a mixture of Bis-GMA, TEGDMA, UDMA, and EBADMA resins reinforced with 60 and 78 wt.% ceramic filler, respectively. It was demonstrated that liquid rubber addition significantly increased the fracture toughness by 9% for flow type and 8% for condensable composite. The influence of liquid rubber on flexural strength was not statistically significant. The addition of the toughening agent significantly reduced Young’s modulus by 7% and 9%, respectively, while increasing deformation at breakage. Scanning electron microscopy (SEM) observations allowed to determine the mechanisms of toughening the composites reinforced with ceramic particles. These mechanisms included bridging the crack edges, blocking the crack tip by particles and dissipation of fracture energy by deflection of the cracks on larger particles. The degree of conversion increased after modification, mainly due to a decrease in the matrix resin viscosity. It was also shown that all dental materials were nontoxic according to ISO 10993-5, indicating that modified materials have great potential for commercialization and clinical applications.

## 1. Introduction

Light cured polymer matrix composites reinforced with ceramic particles used in dentistry are well known since the 1970s [1]. The polymer matrix is commonly a blend of dimethacrylate resins, whereas reinforcement is a mixture of particles, usually of various sizes and chemical composition. These components are supplemented with inhibitors, stabilizers, initiators, pigments, etc., guaranteeing the desired functional and aesthetic features of the final composite. As a result of development of hybrid filling techniques and nanotechnology, the properties of dental composites such as wear resistance, aesthetics, and reliability have been significantly improved [2]. However, an adverse effects of polymerization shrinkage [3] and insufficient mechanical properties of composites such as fracture toughness [4] still remain.

There are many methods widely applied to increase the mechanical properties of dental composites, including the use of round shaped particles [5], whiskers [6], or glass fibers [7]. A promising method for reducing the polymerization shrinkage of the composites and improving their fracture toughness is to modify the resins with rubber [4,8,9]. Different rubbers were used as a toughening agent, for example, low molecular weight butadiene [10] and butadiene–styrene based rubbers [4]. The latest work showed toughness enhancement of the resins for dental applications using polybutadiene/Bis-phenol A copolymers [4]. A good solubility of a methacrylate-terminated poly(butadiene–acrylonitrile–acrylic acid) terpolymer in bisphenol A−glycidyl methacrylate (Bis-GMA) resin also resulted in significantly improved fracture toughness and lowered water sorption of the resin system for dental applications [9]. Most authors examined the toughening with the liquid rubber in the concentration range of 5–20% by weight, with a significant increase in fracture toughness achieved at a concentration of 5–10% wt. of rubber [9,11]. However, the addition of liquid rubber in this amount may negatively affect the Young’s modulus value [12,13]. For this reason, it is necessary to optimize the quantity of toughening agent depending on the intended application.

The articles published so far concern mainly toughening of the resins systems [4,9] without any reinforcement. The question arises, what is the impact of the rubber phase together with ceramic reinforcement on the toughening of the dental composites and other mechanical and physiochemical properties. Furthermore, this kind of modification of the polymer matrix may negatively affect cytocompatibility of the resultant materials. Therefore, the aim of this work was to determine the effect of applied modification of resin-based dental composites on their toughness, elasticity, degree of conversion, viscosity, and cytotoxicity. Importantly, to the best of our knowledge, this is the first study on the toughening and fracture mechanisms of ready-to-use dental composites modified with liquid rubber.

## 2. Materials and Methods

### 2.1. Fabrication of Dental Composites

**Figure 1:**
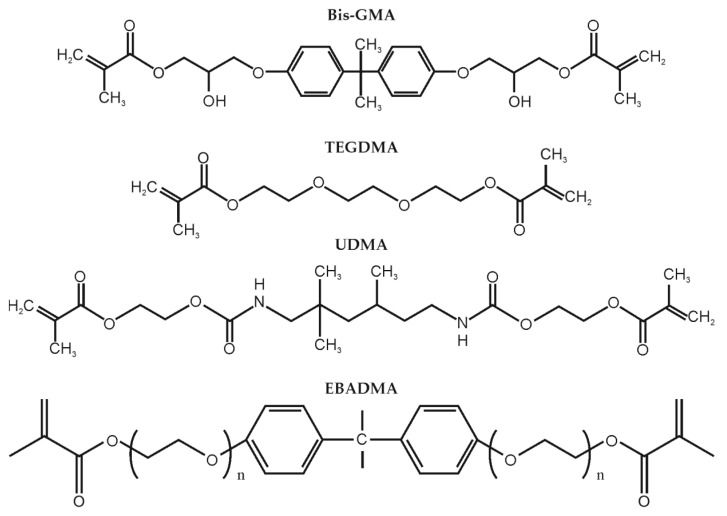
Molecular structure of monomers used in tested composites.

Two commercial composites: Flow-Art and Boston (Arkona Laboratorium Farmakologii Stomatologicznej, Niemce, Poland) were used for testing and modification, the flow (of low viscosity) and the packable type (in the form of dense paste), respectively. Both groups contained the basic and the modified material. The matrix was a mixture of four dimethacrylate resins: Bis-GMA (CAS 1565-94-2), triethylene glycol dimethacrylate (TEGDMA, CAS 109-16-0), urethane dimethacrylate (UDMA, CAS 72869-86-4) and ethoxylated bisphenol A dimethylacrylate (EBADMA, CAS 41637-38-1. (Figure 1). Each resin was purchased from Sigma-Aldrich (Saint Louis, MO, USA). Composition of the mixture was completed by addition of photoinitiator, stabilizer, and inhibitor. Both types of composites contained the same ceramic filler which was a mixture of Ba-Al-B-Si glass, pyrogenic silica, and titanium dioxide. The flow type composite contained 60% ceramics by weight of polymer matrix, while packable composites contained 78% wt. of the reinforcement. Differences occurred in the composition of resins, such as different weight fraction of individual components and different weight fractions of reinforcement particles. In flow type composites (F and FM) one type of the reinforcement particles had a slightly larger size. The exact amounts of ingredients and their composition cannot be revealed as they were patented by the manufacturer (Arkona).

**Figure 2:**
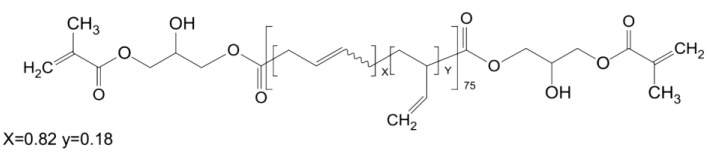
Chemical structure of methacrylate-functional liquid synthetic rubber used as a toughening agent.

The modification was made by addition of 5% by weight (of resin) of a liquid poly(acrylonitrile-co-butadiene) copolymer Hypro^®^ 2000X168LC VTB (vinyl terminated butadiene) (CAS 68649-04-7, CVC Thermoset Specialties, Moorestown, NJ, USA) having reactive functional groups (VTB) capable of chemical bonding to dimethacrylate resins (Figure 2). What is important, liquid rubber used in the research does not contain free acrylonitrile, which may be carcinogenic [14]. Both the composition and the method for the production of liquid rubber modified composites were claimed in the Polish patent application no P.427219 (Light-curing dental composite modified with liquid rubber and the method for its production). The following material designations were adopted: F—flow type (Flow-Art), B—packable type (Boston) and FM and BM—modified F and B composites, respectively.

### 2.2. Mechanical Properties

Fracture toughness *K_Ic_* was determined based on ASTM E 399-20 standard [15] using notched bent samples. According to the above, the transverse dimensions of the samples should meet the condition 1 ≤ *W/B* ≤ 4, where *W* is the height and *B* is the width of the sample. The notch depth *a*, measured after sample break, should be in range *a* = (0.45–0.55) *W*. The following sample dimensions were determined as 15 × 2.2 × 2.2 mm (length, height and width, respectively) so that the span could meet the condition *L* = 4 *W* its length was set at 8.8 mm. The notch was made by placing a 17° steel blade in a mold. Samples were polymerized using led lamp of irradiance 1350 mW/cm^2^ during 20 s on each place covered by the tip of the optical fiber. The tip of the optical fiber was placed directly on the Mylar foil by which the sample was covered. After polymerization specimens were stored in distilled water in the dark for 24 h prior to testing. The tests were carried out on an Autograph AG-X Plus testing machine (Shimadzu) at a traverse rate of 0.5 mm/min, measuring the maximum force *p* at sample breakage. The fracture toughness *K_Ic_* was calculated according to the formula:

(1) $$K_{Ic}=\frac{3\sqrt{\frac{a}{W}}\left[1.99-\frac{a}{W}\left(1-\frac{a}{W}\right)\left(2.15-3.93\frac{a}{W}+2.7{\left(\frac{a}{W}\right)}^{2}\right)\right]PL}{2\left(1+2\frac{a}{W}\right){\left(1-\frac{a}{W}\right)}^{\frac{3}{2}}BW^{\frac{3}{2}}}$$

The flexural strength *σ_f_* and the Young’s modulus was measured using a 3-point bending test. According to ISO 4049:2019 standard [16], the specimens had dimensions of 25 × 2 × 2 mm and the span *L* in this case had a value of 20 mm. The details for specimen preparation were identical to those for *K_1 c_*. The flexural strength was calculated using the formula:

(2) $$\sigma_{f}=\frac{3PL}{2BW^{2}}$$

The Young’s modulus values were calculated according to the ASTM D79017– [17] standard, using the formula:

(3) $$E=\frac{Lm^{3}}{4BW^{3}}$$

where: *m*—slope of the tangent to the initial straight-line portion of deflection, [N/mm].

The flexural strain at breakage *ϵ_f_*, which represents flexibility, was also determined according to the standard mentioned above, using the formula:

(4) $$\epsilon_{f}=\frac{6\delta W}{L^{2}}\times 100\;\left[\%\right]$$

where: *δ* is the maximum deflection at breakage, [mm].

Ten measurements were taken for each material in each case of the test. 

### 2.3. SEM Analysis

After fracture toughness tests scanning electron microscopy (SEM) analysis was performed (Nova NanoSEM 450, FEI Company, Hillsboro, OR, USA) in order to reveal toughening mechanism. Observations were made in a low vacuum mode. 

### 2.4. Degree of Conversion

The degree of conversion (DC) measurements were accomplished using the Fourier Transform Infrared Spectroscopy (FTIR) with Attenuated Total Reflectance (FTIR–ATR). The FTIR–ATR spectra were collected using Hyperion 3000 microscope (Bruker, Billerica, MA, USA) equipped with the MCT detector (Bruker, Billerica, MA, USA) and 20× ATR objective with Ge crystal, attached to Vertex 70 IR-spectrophotometer (Bruker, Billerica, MA, USA). Spectra were measured with 64 scans at 4 cm^−1^ spectral resolution, within the spectral range of 4000–670 cm^−1^. Monomer conversion was calculated using standard methods [18] that evaluated changes in the ratios of aliphatic-to-aromatic C=C absorption peaks (1636 cm^−1^/1608 cm^−1^) in the uncured and cured states obtained from the infrared spectra. The spectra were then analyzed using OPUS 7.0 software (Bruker, Billerica, MA, USA) to obtain the value of 1638 cm^−1^ and 1609 cm^−1^ bands intensity relative to the local baseline [19,20]. The DC value was calculated according to the formula:

(5) $$DC=\frac{1-R_{polim}}{R_{0}}\times 100\;\left[\%\right]$$

where: *R_polim_* and *R*_0_ concerns the data obtained for polymerized and non-polymerized composite sample, respectively, and *R* = height of 1638 cm^−1^ band /height of 1609 cm^−1^ band. The methodology for determining the peak height is shown in Figure 5a.

Ten measurements were taken for each material (*N* = 10). Samples were conditioned in the laboratory for 24 h prior to testing to obtain the same temperature.

### 2.5. Viscosity

The rheology measurements were performed using the HAAKE Rotovisco RT20 rheometer (Thermo Fisher Scientific, Waltham, MA, USA) with 20 mm parallel steel disk and plate geometry with a gap size of 0.2 mm and 1 mm for flow type and packable composites, respectively, according to the methodology presented in [21]. After confirming that the residual vertical stress disappeared, a sinusoidal vibratory shear strain input signal of frequency ω = 0.1–100 rad/s was applied to the plate. The temperature of tested materials was 22 °C. Five measurements were made in each case which allowed to calculate the dynamic viscosity. 

### 2.6. Cytotoxicity Evaluation According to ISO 10993-5

In vitro cell culture experiment was performed using normal human skin fibroblast cell line (BJ, American Type Culture Collection). The BJ cells were maintained in Eagle’s Minimum Essential Medium (EMEM) medium (ATCC-LGC Standards, Teddington, UK) with the addition of 10% fetal bovine serum (FBS, Pan-Biotech GmbH, Aidenbach, Bavaria, Germany), 100 U/mL penicillin, and 100 μg/mL streptomycin (Sigma-Aldrich Chemicals, Warsaw, Poland). The cells were cultured in an incubator at 37 °C in a humidified atmosphere of 5% CO_2_ and 95% air. Cytotoxicity of the materials against eukaryotic cells was evaluated by indirect method using fluid extracts of the tested samples. Extraction procedure as well as cytotoxicity test were conducted in accordance with ISO 10993-5:2009 standard [22] as described earlier [23,24]. Briefly, tested materials were placed in a complete culture medium and incubated at 37 °C for 24 h. The ratio between the material surface area and the volume of the extraction vehicle was approximately 1.25 cm^2^/mL. Culture medium incubated at 37 °C for 24 h without tested samples served as a negative control of cytotoxicity (cell viability = 100%), whereas extract of the latex material served as positive control of cytotoxicity. To assess materials’ cytotoxicity, BJ cells were seeded in 96-multiwell plates in 100 μL of complete culture medium (EMEM) at a concentration of 1 × 10^5^ cells/mL (1 × 10^4^ cells/well). Upon 24-h incubation at 37 °C, culture medium was discarded and replaced with 100 µL of 100% and 50% (diluted in culture medium) materials’ extracts. BJ fibroblasts were exposed to the extracts for 24 and 48 h, and then viability of the cells was assessed by colorimetric MTT test as described previously [24]. The experiment was carried out in 3 independent experiments. Cell viability (%) was determined based on the obtained absorbance values and expressed as the percentage of absorbance obtained with the control cells, revealing 100% viability.

Additionally cytotoxicity of the samples was assessed in a qualitative way using Live/Dead Double Staining Kit (Sigma-Aldrich Chemicals, Warsaw, Poland). After 48-h exposure to the extracts of the materials, BJ cells were stained with calcein-AM (green fluorescence of viable cells) and propidium iodide (red fluorescence of only dead cells) and were observed under fluorescence laser scanning microscope, using a two-dimensional scan technique (Olympus Fluoview equipped with FV1000, Olympus Polska Sp. z o. o., Warsaw, Poland).

### 2.7. Statistical Evaluation

The results were statistically evaluated using the unpaired *t test* and Statistica software mainly to indicate the effect of rubber modification (TIBCO Software Inc., Palo Alto, CA, USA). The results obtained with MTT cytotoxicity test were analyzed for statistically significant differences (*p* < 0.05) using one-way ANOVA test followed by Tukey’s multiple comparison test (GraphPad Prism, Version 5.03, GraphPad Software, San Diego, CA, USA).

## 3. Results

### 3.1. Mechanical Properties

**Figure 3:**
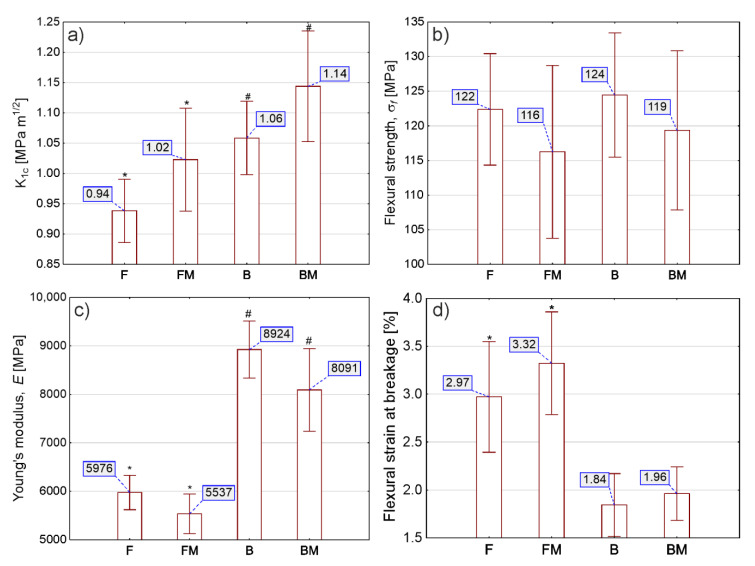
Mechanical properties of the tested materials: (**a**) fracture toughness, *K_Ic_*; (**b**) the flexural strength *σ_f_*; (**c**) the Young’s modulus, *E*; and (**d**) the flexural strain at breakage, *ϵ_f_*. Bars (I) represent standard deviation. Symbol (*) denotes statistical differences between the results obtained with F and FM sample, whereas # denotes statistical differences between the results obtained with B and BM sample.

Mean values for fracture toughness (*K_Ic_*) with standard deviations are shown in Figure 3a. Slight, but statistically significant, differences were obtained for the same type of materials tested (both flow and both packable). The incorporation of the liquid poly(acrylonitrile-co-butadiene) copolymer into the composites’ resin matrix resulted in about 8–9% increase in fracture toughness when compared with the unmodified materials. Increasing the amount of reinforcement from 60% to 78% resulted in increasing by 13% and 12% in fracture toughness, for unmodified and modified composites, respectively.

Modification of tested materials with the liquid rubber slightly decreased the value of flexural strength (Figure 3b). However, results pointed at lack of statistical significance between pairs of results. The condensable composite presented higher value of strength, nevertheless all materials met the requirements of flexural strength included in the ISO standard. 

Results for the Young’s modulus measurement are shown in Figure 3c. Modification of tested composites significantly diminished the value of the Young’s modulus. Increasing the amount of reinforcement from 60% to 78% caused increase of the Young’s modulus value by about 13% and 12%, for unmodified and modified composites, respectively. 

The flexural strain at breakage results are shown in Figure 3d. Modification of flow type (F) composite resulted in a significant increase of strain while for packable type (B) increase was not statistically different. 

### 3.2. SEM Microscopy

**Figure 4:**
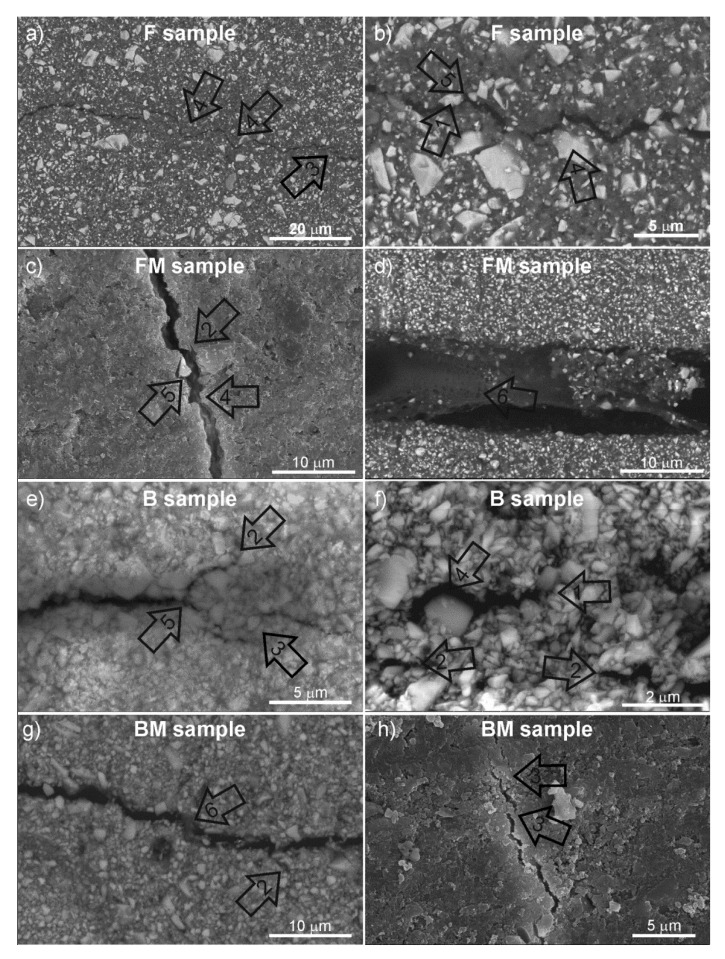
Scanning electron microscopy (SEM) images presenting fractures and toughening features in the tested composites (different magnifications for each pair of images): (**a**,**b**) F sample; (**c**,**d**) FM sample; (**e**,**f**) B sample; (**g**,**h**) BM sample; 1—blocking, 2—secondary cracks, 3—bridging, 4—deflection and bypassing particles, 5—bifurcation, and 6—elastic bridge.

Results of SEM analysis are presented in Figure 4 in order to reveal the fracture mechanisms and toughening features. After the composite was fractured, the testing machine was stopped with a 40% decrease in force to obtain the sample without complete separation, allowing further observation of crack propagation. Both parts of these samples were connected by a narrow strip of material about 0.1 mm wide. This made it possible to observe the crack propagation and evaluate the fracture mechanisms.

All materials exhibited a homogeneous microstructure with the presence of larger reinforcement particles. In the case of the F composite, blocking of the crack tip on the particle and secondary cracks (Figure 4a) as well as the presence of interparticle cracking were visible (Figure 4b). For the FM composite, SEM analysis also revealed blocking of the crack tip on large particles but at the same time the matrix appearance was different from that of the F composite, resembling a viscous liquid surrounding ceramic particles (Figure 4c). Figure 4d explains the toughening mechanism of rubber modified composites. An elastically stretched matrix connecting the two parts was visible between the edges of the crack (bridging mechanism).

Increasing the amount of reinforcement resulted in a stronger break in the crack direction to bypass the particles, including intergranular cracking (Figure 4e) as well as blocking the crack tip (Figure 4f). In the case of modified packable composite (BM), an interparticle cracking with blocking the tip of the crack on the large particle (Figure 4g) and the bridging the cracks edges and blocking the crack tip on flexible areas of the matrix (Figure 4h) were observed.

### 3.3. Degree of Conversion

**Figure 5:**
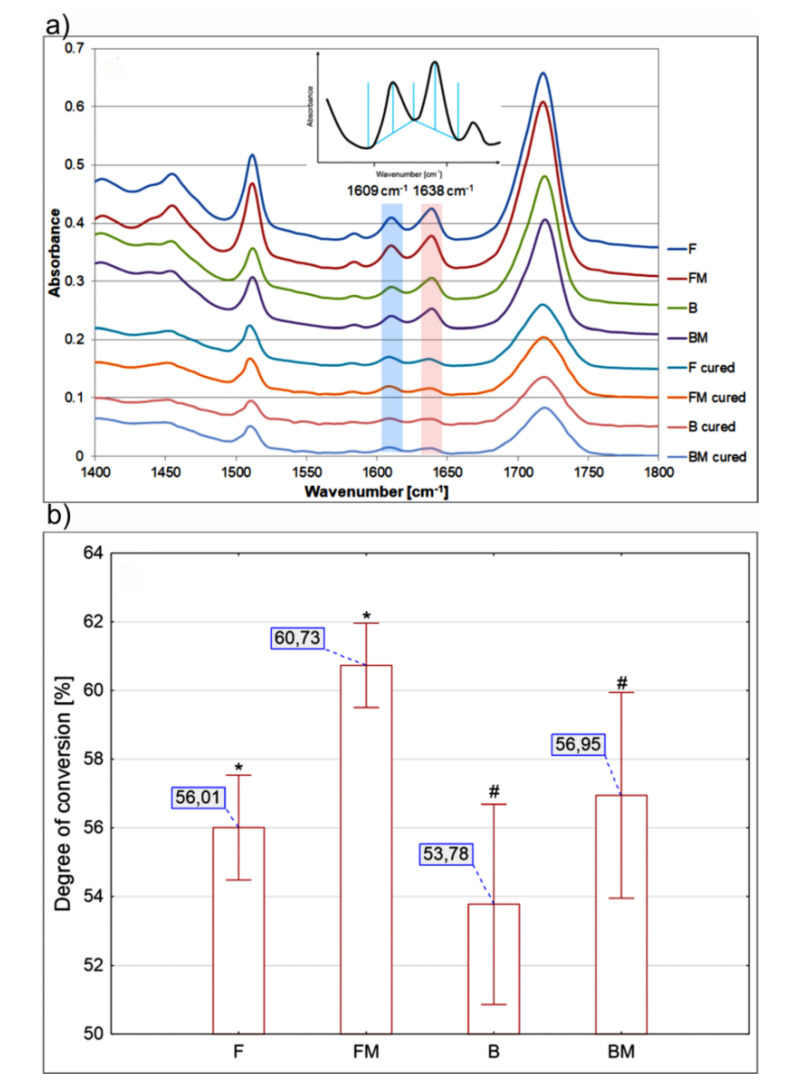
(**a**) Fourier Transform Infrared Spectroscopy (FTIR) with Attenuated Total Reflectance (FTIR–ATR)–ATR spectra together with the method of determining the peak height; (**b**) results of degree of conversion measurements for tested materials. Bars (I) represent standard deviation. Symbol (*) denotes statistical differences between the results obtained with F and FM sample, whereas # denotes statistical differences between the results obtained with B and BM sample.

Results of DC test are presented in Figure 5. Addition of liquid rubber significantly increased the DC value in both types of composites tested. Higher content of ceramics in the materials resulted in lower DC values compared to the corresponding flow type samples.

### 3.4. Viscosity

**Figure 6:**
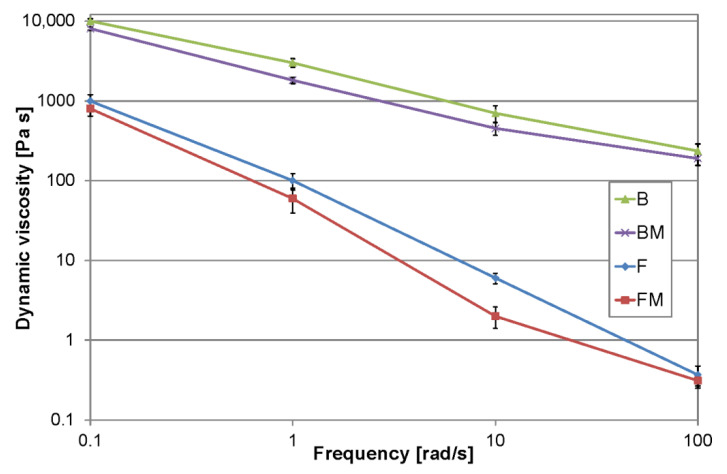
Results of viscosity measurements.

The rheological test revealed statistically significant reduction in viscosity of tested materials after modification with liquid rubber (Figure 6). However, the observed reduction in viscosity was probably the result of increase in the overall monomer content, especially that the liquid rubber used had a viscosity of 100 Pa·s (27 °C). The study aiming to confirm this assumption are currently performed. It is worth to note that viscosity reduction, especially for the FM material, did not cause its leakage from the packaging or loading tip. It is also interesting that the mechanical effect in the case of BM composite (e.g., manual mixing in a package) caused a noticeable reduction in its viscosity, which indicates that shear thinning greatly facilitated manipulation. This is reflected in the graph where the viscosity in the higher frequency range has been significantly reduced. For lower test frequencies, a difference in the order of magnitude between flow and packable materials was noticeable. This difference increased to two orders of magnitude at the highest frequency used in the test.

### 3.5. Cytotoxicity

**Figure 7:**
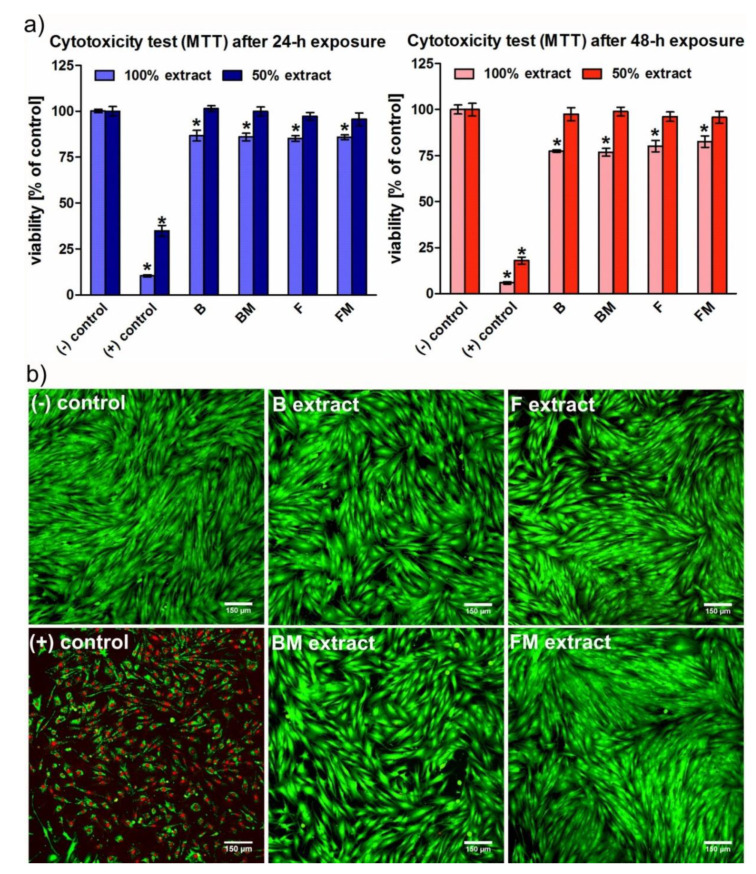
Cytotoxicity of the materials’ extracts assessed according to ISO 10993-5 standard by: (**a**)—MTT test (* significantly different results compared to the negative control of cytotoxicity according to one-way ANOVA test followed by Tukey’s multiple comparison test, *p* < 0.05; (−) control: cells maintained in culture medium, revealing 100% viability, (+) control:cells exposed to latex extract with reduced viability) and (**b**)—live/dead double staining performed after 48-h exposure to the extracts (viable cells—green fluorescence, dead cells—red fluorescence; (−) control—cells maintained in culture medium, revealing 100% viability, (+) control: cells exposed to latex extract with reduced viability; 100× magnification, scale bar = 150 µm).

Cytotoxicity test performed according to ISO 10993-5 showed that all materials were nontoxic to the eukaryotic cells since cell viability upon exposure to the materials’ extracts was above 70% compared to the control cells (Figure 7a). According to ISO 10993-5 recommendations, 100% extract of the medical material that does not reduce cell viability by more than 30% should be considered as nontoxic. After 24-h incubation with the extracts of the materials, fibroblast viability was only slightly reduced to approximately 85%, whereas prolonged exposure to the extracts up to 48 h led to further reduction in cell viability to 77% in the case of B and BM material and to approximately 80–82% in the case of F and FM sample. Cells exposed to the 50% extracts revealed viability near 100% throughout the whole duration of the experiment. Importantly, it was observed that modification of the composites with liquid rubber did not have negative impact on cell viability. Live/dead staining confirmed that extracts of all materials were nontoxic to BJ fibroblasts (Figure 7b). Cells exposed to the extracts had normal morphology, were well spread and showed only green fluorescence, indicating their high viability.

## 4. Discussion

The paper presents mechanical properties and cytotoxicity of the dental composites with different reinforcement content toughened by liquid rubber in relation to the properties of base materials. It was observed that polymerization of composites with addition of toughening agent leads to formation of ‘hard segments’ of polymer backbone, consisting of base resin mixture, and ‘soft segments’ made of polybutadiene. Such soft segments plays an important role in improving fracture toughness and degree of conversion. The presence of liquid rubber was also demonstrated to be beneficial for the viability of eukaryotic cells.

The increase in fracture toughness is reflected in the new strengthening mechanism observed in the SEM microscopy. Soft segments present in the matrix exhibiting viscoelastic behavior undergo elastic and plastic deformations (Figure 4e), thanks to which they are able to absorb the fracture energy. The rubber domains form flexible viscoelastic bridges joining both parts of the crack (Figure 4e,h). This phenomenon in combination with the microcrack formation mechanism results in higher resistance to crack propagation [25]. Furthermore, we observed that the modified flexible matrix absorbs energy through higher deformation (both elastic and plastic), delaying the initiation of cracking. It was confirmed by the value of flexural strain at breakage which was higher in the case of modified matrix, however without statistical significance in the case of the packable composites (B and BM). The increase in flexibility is probably associated with a decrease in crosslinking density [26]. This explains also higher strain value under stress resulting in a lower value of Young’s modulus.

The main failure mechanism observed was the interparticle crack growth. Heterogeneity in the particle size (many large particles) results in the areas rich in resin that promote crack deflection. In the case of both tested composites, their structure was homogeneous with a small particle size distribution which allowed for crack deflection and bypassing mechanism caused by larger particles. A crack blocking mechanism also appears to operate at the crack tip. Such toughening mechanisms are not as effective as crack bridging [27].

Toughening of rubber-modified composites may also result from a significant increase in the degree of conversion by 8.44% for FM and 5.89% for BM composite. These values correspond to the increase in fracture toughness as a result of the modification. Obtained DC values are in the range of 53.7–60.7% and are close to the values obtained for similar materials by Ozturk et al. [28]. The presence of liquid rubber causes a decrease in viscosity, which facilitates the movement of monomers during polymerization. Thus, for higher viscosities obtained for materials B and BM samples, the degree of conversion is lower. According to available literature, improved degree of conversion can have beneficial effect on reduced release of unreacted cytotoxic monomers [19,29]. Importantly, within this study it was demonstrated that modification of resin-based dental materials with liquid rubber does not have negative impact on viability of human cells, indicating that modified composites may be safely used in dentistry.

However, there are some doubts in the interpretation of the increase in conversion obtained while reducing the density of crosslinking [9] after the addition of liquid rubber. Based on the analysis of the literature and research carried out so far, we can conclude that the liquid rubber reduces viscosity which can subsequently increase the mobility of oligomer molecules, facilitating their rearrangement and then the creation of macromolecules. The liquid rubber does not react or only partially reacts with the resin during polymerization [26]. However, the presence of a rubber phase was observed in the polymerized resin blends (results unpublished yet). Therefore, it appears that relative *DC* growth can be observed rather than actual. An inverse relationship can be expected for crosslinking density according to work published by Pionteck et al. [26]. The crosslinking density is decreased due to the reduction of the resin content in the matrix because of the addition of rubber. Moreover, the presence of a dispersed rubber phase separates reactive oligomers preventing or hindering crosslinking. Since this indicates discrepancies, we are going to explain this phenomenon the in subsequent studies.

## 5. Conclusions

Within these studies, it was demonstrated that liquid rubber addition significantly increases the degree of conversion by lowering viscosity and statistically significantly increases fracture toughness to almost the same extent for both types of composites: flow and packable. Furthermore, obtained significantly higher values of *K_Ic_* for composites with higher ceramics content (B and BM) compared to corresponding flow type materials (F and FM) indicate great impact of the reinforcement presence on the fracture toughness. The addition of the toughening agent significantly reduces Young’s modulus while increasing deformation at breakage. Influence of liquid rubber on flexural strength is not statistically significant. SEM observations have shown that the toughening of composites is accomplished by coupling the crack edges by viscoelastic bridge and in addition blocking the crack tip by particles. In conclusion, performed experiments proved that modification of dental composites with liquid rubber significantly improves their conversion degree and mechanical behavior, maintaining high cell viability. Therefore, modified dental composites have great potential for commercialization and clinical applications.

## 6. Patents

The method for the production of the dental materials was claimed in the Polish patent application no. P.427219.
